# Supplementary material for: Evaluation of Ultra–High–Pressure Sterilization in Terms of Bactericidal Effect, Qualities, and Shelf Life of ‘Xinli No. 7’ (Pyrus sinkiangensis) Pear Juice
Source: Foods. 2023 Jul 18;12(14):2729. doi: 10.3390/foods12142729 (PMC10379016; doi:10.3390/foods12142729)
Supplement: Supplementary file 1 [file foods-12-02729-s001.zip › foods-2460527-supplementary.pdf]

Table S1. Response surface test for ultrasonic-ascorbic acid compounds colour protection technology

Levels	Factors		
	A: Ascorbic acid concentration/%	B: Ultrasonic power/W	C: Ultrasonic time/min
-1	0.12	200	5
0	0.18	300	10
+1	0.24	400	15

Table S2. HS-GC-IMS instrument conditions

Instrument	Programs	Parameters
Automatic headspace sampler	Incubate temperature	40 ℃
	Incubate time	20 min
	Sample size	700 μL
	Injection temperature	85 ℃
	Heating mode	Shaking heating
	Incubate speed	500 r/min
GC conditions	Type of column	MXT-5 column
	Parameters of column	15 m × 0.53 mm × 1 μm
	Column temperature	60 ℃
	time	30 min
	Carrier gas	Ultra-pure nitrogen gas (≥ 99.999%)
	Flow rate	2 mL/min (0-2 min); 2-10 mL/min (2-10 min); 10-100 mL/min (10-20 min); 100 mL/min (20-30 min)
IMS conditions	Drift tube temperature	45 ℃
	Voltage in tube	5 kV
	Drift off gas	Ultra-pure nitrogen gas (≥ 99.999%)
	Drift off speed	150 mL/min
	Ionization mode	Positive ion

Table S3. Sensory evaluation of XL7 juice

Score	Colour	Aroma	Tissue conditions	Taste
9-10	Green or light green	With fresh pear fruit aromas, soft and harmonious	Uniform turbidity without stratification	Unique flavour, sour sweet and delicious
7-8	Yellow or light yellow	Fresh pear aroma is light and not soft	Uniform turbidities with a small amount of flesh slightly stratified	Medium sweet and sour
5-6	Light brown	No fruit aroma, peculiar smell	Much stratification	Inappropriate sweet and sour ratio
1-3	Dark brown	No fruit aroma, severe peculiar smell	Severe stratification	Too sour/too sweet
